# Determination of Haematological Reference Values for Tucúquere (*Bubo magellanicus*) Habiting in Central Chile

**DOI:** 10.3390/ani13193000

**Published:** 2023-09-22

**Authors:** Alejandro Jimenez-Cortes, Sergio Boassi, Hernan Cañon-Jones

**Affiliations:** 1 Núcleo de Investigación Aplicada en Ciencias Veterinarias y Pecuarias, Facultad de Medicina Veterinaria y Agronomía, Universidad de Las Américas, Santiago 7500975, Chilesboassi@udalba.cl (S.B.); 2 Facultad de Ciencias Agropecuarias, Universidad del Alba, Santiago 8320000, Chile

**Keywords:** owls, human–animal interaction, animal health, animal welfare, environmental sustainability

## Abstract

**Simple Summary:**

The tucuquere (*Bubo magellanicus*) is an owl inhabiting the American continent, but its current conservation status is not well known. Species-specific haematological reference values are valuable for diagnosis and prognosis in clinical veterinary and rehabilitation centres. We determined the haematological reference values from 33 samples from tucúqueres taken from zoos and rehabilitation centres in central Chile using standard laboratory techniques and reference values. The mean, standard deviation, maximum, and minimum reference limits were calculated at 95% and 97.5% error. The main results indicate lower values for white blood cell lines and not for red blood cells compared to other genetically closed owls. These results could be explained by the habitat and location and corroborate the need to obtain reference values for different ecological geographical areas. This study provides useful information for conservation medicine and zoos in Chile, but also highlights the importance of carrying out these studies in different geographical areas globally.

**Abstract:**

Tucuquere (*Bubo magellanicus*) is an owl inhabiting Chile, which is classified as a species of agricultural interest, but its current conservation status is not well defined. The determination of previously unknown haematological ranges via laboratory techniques for species analysis is of great importance in the search for diagnoses in clinical veterinary work. Thirty-three samples from healthy tucuquere were obtained from zoos and rehabilitation centres in central Chile and analysed using standard laboratory techniques to obtain reference values that were determined according to the standard recommendations for animal species. The mean, standard deviation, maximum, and minimum reference limits were calculated at 95% and 97.5% error. These results differ from those of other studies, especially in terms of the white blood cells because most of the values were lower than those described for the species. These results could be explained by the habitat and location from which the samples were taken, and they corroborate the need to obtain reference values for different geographical areas. This study provides useful haematological values for use in conservation medicine and zoos and highlights the importance of carrying out these studies in different geographical areas for species of ecological interest.

## 1. Introduction

Chile is a country with a great diversity of environments but with biogeographic isolation for thousands of years, leading to a low number of animal species, unlike in other places. Birds represent the second largest group of vertebrate individuals in the country with 456 species [1], of which 2% (10 species) are endemic and 60% are resident. However, if the Chilean-Argentinian Patagonia zone is considered, the percentage of endemism reaches 60%. Therefore, wild bird conservation in Chile is not only of local importance but also of great global significance [2]. Chile is home to 34 species of birds of prey, 27 of which are diurnal (Order Falconiformes) and 7 are nocturnal (Order Strigiformes) [3]. All wild birds are protected by the provisions of Chile’s Hunting Law No. 19473 of 1996 and the regulations of the Ministry of Agriculture, which prohibit hunting and capture [4].

The tucúquere (*Bubo magellanicus*) is an owl that lives in Chile and is classified as a species at lower risk according to the International Union for Conservation of Nature of the year 2017 [5]. *Bubo magellanicus* is considered to be the largest owl in Chile and a subspecies of *Bubo virginianus*, which is an owl from North America [3]. Today, the scientific name of this species remains debatable, since specialists believe that it would correspond to *Bubo magellanicus*, being a species as such and not a subspecies of *Bubo virginianus*, which is the owl of North America [6]. *B. magellanicus* reaches a length of 50 cm and a wingspan of 1 m; its weight is approximately 850 g, and the female is larger than the male [3]. *Bubo magellanicus* is characterized by its important role as a top predator in the trophic chain. It is considered a bird of prey capable of controlling the natural populations of the species it feeds on, which may be important for conservation and environmental health [7]. Its location is observed in habitats mainly of mountain ravines and forest areas, but it can also be seen in central valleys and on the coast. Its distribution in South America is on the coast of Peru, west of Bolivia and Argentina, in Chile from Arica through the northern desert in watery ravines of the mountain range to Tierra del Fuego and Cabo de Hornos [8]. Its reproduction begins in the month of October, laying no more than three eggs in the recycled nests of other birds of prey. Other places of preference are cracks in rocks of considerable depth and cliff caves. However, recent studies have confirmed that there are nesting sites in houses within larger cities such as Santiago, increasing the chances of human interactions [9]. The chicks hatch in November and birds can live in wildlife for 15 to 18 years, and in captivity for 30 years [8].

Vulnerability in wildlife has reflected a decline in the population due to the fragmentation and reduction of its habitat, accompanied by hunting, abuse, electrocution, and toxicity [10], especially by human expansion into wildlife habitats [11]. Urbanization, agricultural activities, and infrastructure development lead to habitat fragmentation, degradation, and loss, which can result in the displacement of wildlife populations and the destruction of their habitats [12,13]. Human–wildlife conflict is also a major issue, as it can lead to the loss of wildlife populations and damage to human property [14,15]. Furthermore, human activities can increase the risk of zoonotic diseases, which can have devastating effects on both wildlife and human populations [16,17]. The spread of infectious diseases can be facilitated by the movement of wildlife by humans, habitat fragmentation, and outbreeding depression [18].

The determination of haematological ranges via laboratory techniques to perform an analysis of a species is of great importance to evaluate the health of animals in veterinary clinical work [19]. Blood count is a routine examination that has a high priority since it is essential in the confirmation or rejection of pathologies, where the normal parameters may indicate if the animal is healthy or may exclude the possibility of being an asymptomatic carrier of any disease [20,21]. This tool is valuable in the clinic for birds, both for prognosis and treatments, as well as for understanding various pathophysiological situations that affect this owl [22,23]. Haematology is one of the many tools used to assess health status, nutritional deficiencies among populations, or immunosuppression due to stressors in wild populations of raptors [24]. Raptors sit at the top of many food webs, allowing their health status to reflect the overall health of ecosystems [25]. Alterations in haematological variables may indicate changes in habitat quality and food availability, as well as reflect exposure to pollutants or toxins [26]. From the moment that the reference ranges of an avian species are established, it is possible to assess any deviation of blood components in rescued birds from rehabilitation centres, or research, and unravel possible causes that affect health and welfare [21,22,27]. Although avian haematology has begun to be studied, it has not achieved the level seen in mammals and humans [28].

The development of haematology in raptors and birds, in general, is scarce, as it requires obtaining reference values for each species, with an understanding of how physiological and pathological stimuli alter their response [29]. Much research has focused on determining haematological reference intervals, the results of which are available in databases such as ISIS (www.ISIS.org) and LYNX [30]. However, there is little information regarding the normal haematological ranges of Tucúquere in Chile. For this reason, this study aimed to obtain the haematological values of *B. magellanicus* in a healthy state in the Central Zone of Chile.

## 2. Materials and Methods

Samples were obtained from individual birds which were sent to rescue and rehabilitation centres in two regions of central Chile. Five birds were from Rancagua Safari Park, 9 from Unidad de Rehabilitación de Fauna Silvestre from Buin Zoo (UFAS), 8 samples from the National Zoo of Chile, and 11 samples from the Centro de Rehabilitación de Aves Rapaces (CRAR), covering the central zone of Chile. All animals were recovered from the wild due to various types of accidents (mainly road accidents or electric cable accidents). Minimal bird handling was carried out, and birds received veterinary treatment and were allowed to fully recover in individual cages until release with minimal handling and minimal human interaction. Sampling was carried out the day before the birds were released to the wild to verify full recovery and good health. To corroborate optimal health, a full veterinary clinical check-up was carried out, including evaluation of physiological variables such as body temperature, heart rate, and respiratory rate, as well as parameters such as body condition, colour of the mucous membranes, status of skin and feathers, oral cavity, legs, genital area, and presence/absence of ectoparasites. A blood smear evaluation was also carried out to identify the presence of any blood parasites. The birds were identified as adults, and no sex determination was carried out as this species has very little or no sexual dimorphism, and we did not carry out DNA sampling for sex determination. Blood samples were collected between June and August 2018 and processed by a specialised veterinary laboratory, Vetlab. Vetlab is the main veterinary clinical laboratory for zoos, veterinary clinics, and hospitals in Chile. Blood samples (approximately 1 mL) were extracted from the brachial vein using a 23G needle (Nipro Corporation, Osaka, Japan). During the blood extraction process, excessive suction was avoided to prevent haemolysis. Gentle but firm pressure was maintained at the vein puncture site for a couple of seconds to ensure no further bleeding. Blood was deposited in cryovials with EDTA and refrigerated until analysis in the laboratory. Samples were analysed by a private veterinary laboratory using an automatic haematological analyser (BC-3600, Mindray^®^, Shenzhen, China) for red blood count. White blood count was carried via an indirect method using floxine B, which specifically stained heterophils and eosinophils [31]. In brief, (a) an Unopette pipette was filled with blood (25 µL) and mixed for 5 min, after which the initial drops were discarded and the counting Neubauer chamber was loaded, followed by waiting for 5 min; (b) granulocytic leukocytes were counted throughout the reticule on both sides of the counting chamber (heterophils and eosinophils exhibited red-orange staining and appeared round and refractile; (c) finally, total leukocyte (μL) was calculated as (the number of stained cells counted × 1.1 × 16 × 100)/(% heterophils + % eosinophils).

To determine the reference values for *B. magellanicus*, standard recommendations were followed [32,33,34]. Briefly, using the obtained data, the mean, median, standard deviation, typical errors, maximum and minimum reference limits at 95% and 97.5% error, with a 90% confidence interval, and the distribution curves of the species studied were calculated. For this purpose, the free statistical program R version 4.2.1 was used [35].

## 3. Results

The haematological reference values of tucúquere (*Bubo magellanicus*) are shown in Table 1.

## 4. Discussion

Most variations in this study, compared to other studies for reference values, were found in the white blood series. Our results showed lower values for the white cell series compared to previous studies on *B. magellanicus* in different geographical areas. The number of white blood cells changed according to an individual’s life stage, sex, and stress [36]. Handling stress that occurs during capture is considered one of the most extensively studied stimuli that can cause variations in the leukocyte count of birds [37]. Also, another important environmental factor is photoperiod, or the location related to it, which can influence the number of leukocytes in birds [38].

The total white blood count, heterophiles, eosinophiles, and basophiles were lower than those found in Brazil, Chile, and the eastern United States [39,40], and were only similar to those found in Canada and southwest central United States [40].

However, the lymphocyte count was consistently higher than those reported in the same studies [39,40,41].

Thrombocyte levels in this study were lower than those reported in one study in Brazil [42], but higher than those in another study in the same country by the same authors [43]. In the same studies in Brazil [42,43], monocytes were found to be lower than those in the present study.

A possible explanation for our results is the sample size, which is another important factor when establishing reference interval values, and it is recommended that it is as large as possible [43]. In this respect, previous studies [39,41,43] had a much smaller sample size (less than five individuals), so these values might not be representative of the species. The results of our work are obtained from a larger number of individuals than those of the previously mentioned studies, so they may be closer to those of the species.

Also, it is known that haematological values may be affected by several factors, such as stress, sex, and body size. Stress can alter the leukocyte count [37], thereby producing leucocytosis, heterophily, lymphocytosis, or lymphopenia [43]. Previous studies were conducted on rescued birds with known trauma [39,41,43] and possible stress. The samples taken in our study were collected at the end of the rehabilitation process from clinically healthy animals, and this condition may explain the lower results in the present study.

Also, sex might be a confounding factor and the sex of wild birds can indeed be challenging in many species as they often lack external physical differences between males and females that are easily visible to human observers. Unlike some mammals, where males and females may have distinct size, colour, or ornamentation differences, many bird species show minimal or no obvious external sexual dimorphism. In our study, we did not determine sex as this species does not show marked sexual dimorphism. Future studies should evaluate this variable using new DNA technologies.

Red blood cells, haematocrit, haemoglobin, MCH, MCHC, and MCV reference values were in accordance with all previous studies on *B. magellanicus*, *B. virginianus,* or *Strigiforms* [39,40,41,42,43]. These results also suggest that the birds sampled were not stressed, and the values from the white blood series are representative of the normal conditions of tucúqueres.

## 5. Conclusions

This study determined the haematologic reference values for tucúquere in central Chile. The results may be useful for rescue and rehabilitation centres, allowing veterinarians, biologists, and conservationists to determine the health and welfare status at any stage of the rehabilitation process (entrance, during, and at release). This can be performed whenever a blood sample is taken and by comparing if values are within the expected ranges for the species. Also, the study highlights the importance of determining a haematological reference for each ecological and geographical condition.

## Figures and Tables

**Table 1:** Haematological reference values of tucúquere (*Bubo magellanicus*) in central Chile (*N* = 33).

Variable	Mean ± SD	Min–Max	Median	Lower 90%CI	Upper 90%CI
RBC (10^6^/μL)	1.8 ± 0.3	0.9–2.6	1.78	1.7	1.9
Haematocrit (%)	36.5 ± 6.2	17.0–50.0	39.0	34.7	38.4
Hb (g/dL)	11.5 ± 1.9	5.9–16.0	12.0	10.9	12.1
MCH (pg/cell)	62.2 ± 9.5	44.0–91.4	60.6	59.4	65.1
MCHC (g/dL)	31.7 ± 1.5	30.0–36.2	32.0	31.2	32.2
MCV (µ/fL)	195.4 ± 21.2	156.0–236.0	186.0	189.2	201.7
WBC (μL)	14,036 ± 8504	3200–52,200	11,100	11,528	16,544
Heterophiles (μL)	9868 ± 6061	1728–35,496	8008	8081	11,656
Eosinophiles (μL)	206 ± 163	0–702	184	158	255
Basophiles (μL)	0	0–0	0	0	0
Lymphocytes (μL)	3314 ± 3100	675–15,015	2376	2400	4229
Monocytes (μL)	647 ± 562	148–3132	462	481	812
Thrombocytes (10^3^/μL)	186 ± 100	20–380	188	157	216
Rate H/L	4.0 ± 2.1	0.4–9.5	4.1	3.4	4.7

Note. The CI of the mean assumes that sample means follow a t-distribution with N − 1 degrees of freedom. RBC: red blood count; MCH: mean corpuscular haemoglobin; MCHC: mean corpuscular haemoglobin concentration; MCV: mean corpuscular volume; WBC: white blood count; H/L ratio: heterophil/Lymphocytes ratio.

## Data Availability

Data may be distributed and sent by request.
